# Supplementary material for: Shared Treg dysregulation underlies psoriasis vulgaris and atopic dermatitis through the PHF19-PRC2 epigenetic axis
Source: Front Immunol. 2026 Aug 20;17:1932788. doi: 10.3389/fimmu.2026.1932788 (PMC13538097; doi:10.3389/fimmu.2026.1932788)
Supplement: Supplementary file 3 [file DataSheet3.zip › Additional files/Additional file 4/55.HACD4.leaveoneout.pdf]

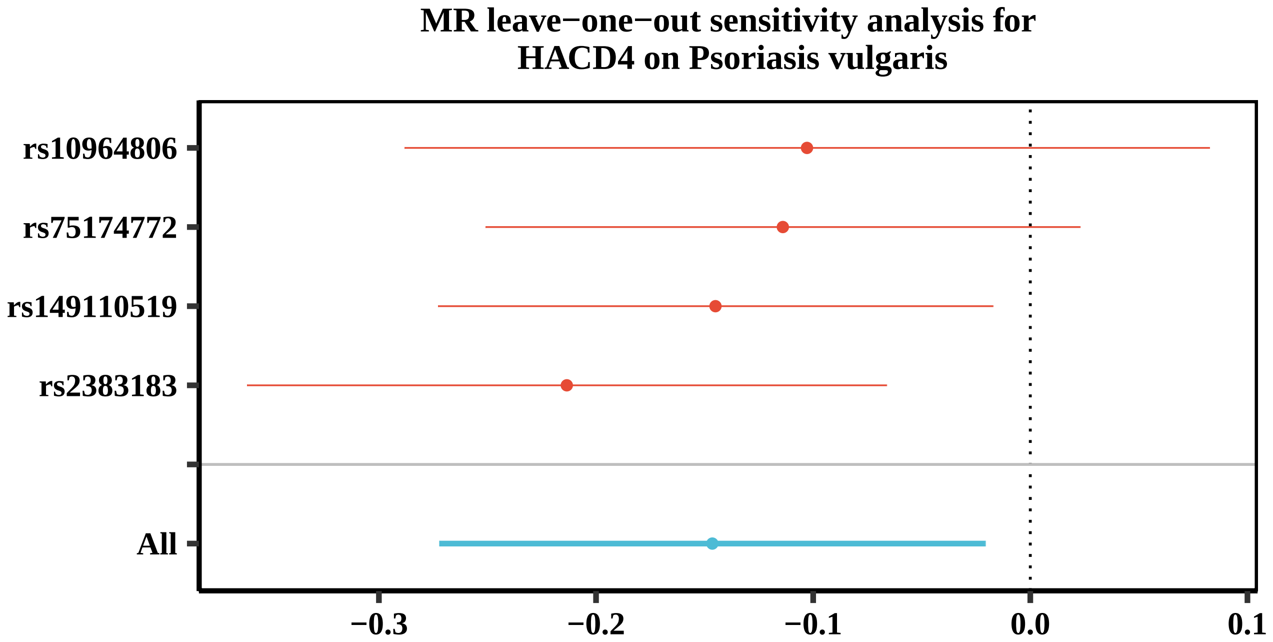
MR leave−one−out sensitivity analysis for
HACD4 on Psoriasis vulgaris
rs10964806
rs75174772
rs149110519
rs2383183
All
−0.3
−0.2
−0.1
0.0
0.1
